# 

**Published:** 1870-05

**Authors:** 


### THE AMERICAN JOURNAL OF DENTAL SCIENCE,

EDITED BY

F. J. S. GORGAS, M. D., D. D. S.

VOL. 4.—THIRD SERIES.

Surgeon Genl's Office LIBRARY. 36914

BALTIMORE:
SNOWDEN & COWMAN.
LONDON:
TRUBNER & CO., 60 PATERNOSTER ROW.
1871.

WI AM 45M

3375c

### INDEX TO VOL. IV.--THIRD SERIES.

A Case in Surgery a Hundred Years Ago. - - - - 474
A Gold Medal. - - - - - - - - - - - 383
A New Anæsthetic. - - - - - - - - - - 426
A Singular Tumor. - - - - - - - - - 335
A Snake in the Stomach. - - - - - - - - 42
Abbott, Frank, D. D. S. - - - - - - - - - - 361
Aconite and Muriate of Ammonia in Neuralgia. - - 238
Address before the Tennessee Dental Association. - - 223
Adhesive Gold Foil. - - - - - - - - - 124
Adulteration of Vinegar. - - - - - - - - - 522
Allen, John, D. D. S. - - - - - - - - - 399
Allen's Continuous Gum Work. - - - - - - - - 73
Almost Complete Ossification of the Human Body. - - 40
Aluminum as a Base for Artificial Teeth. - - - - 159
Aluminum Plates. Method of Casting, - - - - - 573
Alveolar Processes and Gums. Wasting of, - - - - 110
Amalgam. - - - - - - - - - - - - 315
Amalgam for Filling Carious Teeth. - - - - - 172
American Academy of Dental Science. - - - - - 355
American Dental Association. - - - - - 144, 197, 251
Among the Dentists. - - - - - - - - - - - 284
Amputation Extraordinary. - - - - - - - - 92
An Operation on the Lower Jaw, - - - - - - - 170
Anæsthesia, - - - - - - - - - - - 181
Anæsthetics. Administration of, - - - - - - 161
Anæsthetics. New, - - - - - - - - - - 236
Anderson. Edw. W., D.D.S. - - - - - - - - 550
Animalcules on the Teeth. The Effect of, - - - - 289
Antrum. Disease of, - - - - - - - - - - 550
Antrum. Inflammation of, - - - - - - - - 454
Are the Mineral Acids formed in the Mouth. - - - - 327
Asthma. - - - - - - - - - - - - 524
Atkinson. W. H., D.D.S., M.D. - - - - - - - 110
Bad Effects of Chloral. - - - - - - - - 422
Bakers. W. C., M.D. - - - - - - - - - - 412
Baltimore College of Dental Surgery, - - - - 335, 525
Bean. James Baxter, D.D.S. Death of, - - - - - 287
Beers. W. Geo. L.D.S. - - - - - - - 75, 172, 502, 553
Bibliographical Notices. - - - - - 238, 282, 334, 427
Arthur's Home Magazine. - - - - - - - 428

Deformities of the Mouth. J. Oakley Coles. - - - 374
Descriptive Catalogue of New Sydenham Society's Atlas of Skin Diseases. - - - - - - - - - 335
Manual on Nitrous Oxide Gas. F. R. Thomas, D.D.S. 238
Pathologie Der Zahne. - - - - - - - - - 427
Scribner's Monthly. - - - - - - - - 429
Scientific American. - - - - - - - - 427
The Art Review. - - - - - - - - 430
The American Agriculturist. - - - - - - - 429
The Children's Hour. - - - - - - - - 428
The Bright Side. - - - - - - - - - 428
The Herald of Health. - - - - - - - - 428
The Manufacturer and Builder. - - - - - - 428
The New York Observer Almanac, for 1871. - - 429
The Medical Times. - - - - - - - 282
The Physician's Visiting List for 1871. - - - - 335
Transactions of Illinois State Dental Society. - - 238
Twelfth Annual Report of Chicago Eye & Ear Infirmary. 427
Webster's Dictionary. - - - - - - - - 238
Bichloride of Methylene, - - - - - - - 44
Bill, J. H., M. D. - - - - - - - - - - 373
Brewster, Dr., Death of, - - - - - - - - - 432
Borax. - - - - - - - - - - - - 521
Bowdoin. W. L., D.D.S., Death of, - - - - - 153
Bromide of Potassium in Dentition. - - - - - 136, 137
Bromoform, Bromal. - - - - - - - - - - 236
Bromwell, J. R., D.D.S. - - - - - - - - 14, 49
Brown, J. P, H., D.D.S. - - - - - - - - 241
Briggs, Prof. - - - - - - - - - - - - 420
Buckingham, T. L., D.D.S., M.D. - - - - - - - 509
Carbolic Acid as an Antiseptic and Prophylactic. - - - 168
Carbolic Acid. Uses of, - - - - - - - - - - 93
Carbolic Acid in Carbuncle. - - - - - - - - 525
Carbolic Acid as a Local Anæsthetic, - - - - - - 373
Carbolic Acid Soap. - - - - - - - - - - 381
Carbolic Collodion. - - - - - - - - - - - 331
Carothers. T. D., M.D. - - - - - - - - - - 463
Case of Tumor of Soft Palate. - - - - - - - - 559
Cells with Fibres in Dentine at Junction of Enamel and Cementum. Discovery of - - - - - - 1
Cleft Palate, Case of - - - - - - - - - 116
Chase. H. S., D.D.S., M.D. - - - - - - - 327, 454
Charcoal. - - - - - - - - - - - - - 91
Chloral. Test for the Purity of, - - - - - - 571
Chloral Hydrate. - - - - - - - 186, 412, 475, 506
Chloral Hydrate. Bad Effects of - - 422, 556
Chloroform. Death from, - - - - - 39
Chloroform in Infantile Convulsions. - - 22

Chloroform. Notes on, - - - - 97
Chloroform, Pro and Con. - - - - 119
Clark. F. Y., D.D.S. - - - - 145
Coating of the Tongue. - - - - - 432
Cobb. S. J., D.D.S. - - - - 223
Colton. G. Q. - - - - - - - - - 181
Colton. John J., M.D. - - - - - - - - 154
Complicate Fillings. - - - - - - - - 246
Convulsions. - - - - - - - - - - 88
Crouse. J. N. Dr. - - - - - - - - - 350
Cushing. G. H., D.D.S. - - - - - - 299, 437, 492
Cutler. S. P., D.D.S., M.D. - - - - 193, 218, 394, 434
Cystic Tumor of the Lower Jaw. - - - - - - - 137
Cystic Tumor of the Lower Jaw with Giant-celled Elements. 376
Dangers of Snuff Taking. - - - - - - - 520
DeRosset. M. J., M.D. - - - - - - - - 97
Dean. M. S., D.D.S. - - - - - - - 246
Death from Chloral Hydrate. - - - 522
Death from Chloroform. - - - - - 39, 139, 379, 423
Death following Application of Creasote to a Tooth. - - 43
Death from Inhalation of Ether. - - - - - - 523
Deformity of the Mouth. - - - - - - - - 324
Dental Chemistry, - - - - - - - - - - 399
Dental Decay. Prophylaxis or Prevention of - - - 102
Dental Practice. Notes from - - - - - - - 151
Dental Items. - - - - - - - - - 383, 430, 572
Dental Neuralgia. - - - - - - - - - - 218
Dental Pulp. Diseases and Treatment of - - - - - 145
Digestion. Recent Views on - - - - - - - 14, 49
Doses for Hypodermic Use. - - - - - - - - 45
Double Hare Lip. - - - - - - - - - 465
Duty Incumbent upon Dentists to Attend Associations. - 453
Dyspepsia as a Cause of Consumption. - - - - - 183
Dyspepsia—its Causes and Cure. - - - - 86
Eames, W. H., D.D.S. - - - - - 357
Effect of Animalcules on the Teeth. - - - - - 289
Effect of the Use of the Sewing Machine. - - - - 475
Encysted Tumors of the Ducts of the Submaxillary. - - 227
Ergot to Arrest Excessive Perspiration. - - - - 91
Excision of a Portion of the Lower Jaw. - - - - - 516
Extraction of Artificial Teeth and Metallic Plate from the Œsophagus. - - - - - - - - - - - 479
Exostosis. - - - - - - - - - - - 567
Erratum. - - - - - - - - 576
Facial Neuralgia. - - - - - - - - - - 140
Fatal Tooth Pulling - - - - - - - - - - 39
Fibro-Serous Cyst of Jaw. - - - - - - - - 569
Filling Teeth. - - - - - - - - 76, 485, 572

Flexible Edge for Rubber Plates. - - - - - 452, 553
Foster. J. H., M.D. - - - - - - - - - - 540
Function of the Prostate Gland, - - - - - - - 569
Gargle of Iodine. - - - - - - - - - - - 188
Garretson. J, E., M.D., D.D.S. - - - - 465, 470, 513
Germinal Matter. - - - - - - - - - - - 385
Gold Plates with Rubber Attachments. - - - - - 357
Gibbons. H., M.D. - - - - - - - - - - 321
Gross. Prof. Samuel D., M.D. - - - - - 320, 376
Guillois' Cement for Temporary Fillings, - - - - - 382
Gums, Inflammation of, - - - - - - - - - 241
Hamilton. Prof. Frank, M.D. - - - - - - - - 227
Harris. Edw. N., D.D.S. - - - - - - - - 355
Harris' Principles and Practice of Dental Surgery. - - 95
Harriman. Geo. B., D.D.S. - - - - - - 1, 289
Harvey. L. F., M.D. - - - - - - - - - 79
Hasbrouck. T., D.D.S. - - - - - - - - - 73
Health. - - - - - - - - - - - - 523
Health Restored by Extraction of Diseased Teeth. - - 151
Heavy Foils and Heavy Mallets. - - - - - - - 299
Hiccough. - - - - - - - - - - - - 237
Histology of the Dental Tissues. - - - - - 59, 95, 276
Hodgkin. James B., D.D.S. - - - - - - - 57, 345
Hoffer. J. Z., D.D.S. - - - - - - - - - 5
How to Cure a Cold. - - - - - - - - - - 41
How to Detect Adulterations in Candy. - - - - - 92
How to Walk and Stand. - - - - - - - - - 431
Hunter. E. L., D.D.S. - - - - - - - - - 485
Hygienic Information. - - - - - - - - - 377
Hydrate of Chloral. - - - - - - 39, 412, 475, 506
Hydrate of Chloral and Morphia in Tetanus. - - - - 476
Hypetrophy of Roots of a Superior Molar Tooth. - - 567
Identity of the White Corpuscles of the Blood with Salivary Pus and Mucous Corpuscles. - - - - - - 364
Immobility of the Jaws. - - - - - - - - - 420
Impressions of Difficult Partial Cases. - - - - 575
Inebriation Hereditary. - - - - - - - - - 187
Inflammation of the Antrum. - - - - - - - - 454
Inflammation of the Dental Pulp, - - - - - 529
Inflammation of the Gums. - - - - - - - - 241
Inflammability of Pyroxyline. - - - - - - 573
Influence of Diet on the Composition of Bone. - - - 332
Influence of Sewing Machines on Health. - - - - 44
Iodal. - - - - - - - - - - - - - 236
Iodide of Potassium in Periostitis. - - - - - - - 380
Iodine Gargle. - - - - - - - - - - - 188
Irregularities of the Teeth. - - - - - - - - 34
Iron Retorts for Nitrous Oxide. - - - - - - - - 152

Jack. Louis, D.D.S. - - - - - - - - 124
Jones. Henry C., D.D.S. - - - - - - - 481
Judd. H., M.D., D.D.S. - - - - - - - - 276
Keesee. Geo. F., D.D.S. - - - - - - - 354
King. Kelburne, M.D., F.R.C.S. - - - 559
Knapp. J. S., D.D.S. - - - - - - - 413
Lactic Acid in Croup. - - - - - - - 90
Lancing the Gums in Dentition. - - - - - - 321
Lancing the Gums. Indications for, - - 565
Light vs. Heavy Gold Foil and Crystal Gold. - - - 361
Lithotrity. - - - - - - - - - 41
Loosening of the Teeth. - - - - - - - 79
Loss of Speech after Chloroform. - - - - - 380
Ludwig. James H., M.D., D.D.S. - - - - - - 529
Mackintosh. David, M,D., L.R.C.S. - - - - - 22
Manufacture of Ozone. - - - - - - - - 424
Maunder. C. F., M.D. - - - - - - - - 170
McDougall. S. J., D.D.S. - - - - - - - - 452
McLain. A. F., M.D., D,D.S - - - - - 102
Mechanical Dentistry. - - - - - - - - 5
Mental Strain as a Disease. - - - - - - - 283
Mercury in Vulcanized Rubber Plates. - - - - 57
Mercurial Salivation. - - - - - - - - 293
Methylic Ether as an Anæsthetic. - - - - - - 88
Microscopy of a Tooth. - - - - - - - - 434
Microscopy of a Deciduous Tooth. - - - - - 193
Modern Surgery. - - - - - - - - - 524
Monument to Dr. Horace Wells. - - - - - 47
Morgan. W. H., D.D.S. - - - - - - - 406
Nashville Meeting. - - - - - - - - - 238
Necrosis of the Lower Jaw from Undeveloped Teeth. - 312
Neuralgia Pill. - - - - - - - - - - 381
Neuralgic Pill. Osborn's, - - - - - 572
Neuralgia of Jaw. - - - - - - 570
Neuralgia of the Jaw Bone. - - - - - - 320
New Operation on the Lower Jaw. - - - - - 41
New Orleans Dental College. - - - - - - 526
Nitrous Oxide Gas. - - - - - - - - - 345
Noel. H. R., M.D. - - - - - - 59, 337, 385
Notes on Chloroform. - - - - - - - - - 97
Notes on Dental Practice. - - - - - - - 151
Obituary Notices. . . . . . . 240, 287, 432
Operative Dentistry. Report on, . . 437, 492
Operative Dentistry. W. H. Morgan, D.D.S. . . 406
On the Use of Chloroform in Infantile Convulsions. . 22
Ossified Pulp. . . . . . . . . . 193
Oxalic Acid for the Sting of a Bee. . . . . 94
Oil of Peppermint as a Local Anæsthetic. . . . 476

Painless Knife. Richardson's, . . . . . 93
Paralysis of Side of Face. . . . . . . 470
Periodontitis. . . , . . . 369
Physiological Action of Protoxide of Nitrogen. . . 154
Physiological Effect of Muscular Exercise. . . 425
Pneumatic Bur Engine. . . . . . . 144
Poisoning from Chloral Hydrate. . . . . 556
Poisoning by External Use of Carbolic Acid. . . . 475
Poisoning by Worm Lozenges. . . . . 381
Poisonous Indelible Ink. . . . . . 476
Prevention of Iron Rust. . . . . 184
Professional Shortcomings. . . . . , 540
Prophylaxis or Prevention of Dental Decay. . . . 102
Pernicious Effects of Mercury Prevented by Sodium. . 473
Pyroxyline Base for Artificial Dentures. . . 502, 573
Rapidity of Mental Transmission in a Nerve. . . 477
Ready Rubber. . . . . . . . 478
Recent Views on Digestion. . . . . . 14, 49
Recipe for Burns. , . . . . . 89
Reed. Thomas N., D.D.S. Death of, . . . . . 240
Removal of Articular Extremity of Lower Jaw with Restoration of Motion. . . . . . . . 380
Report on Operative Dentistry. . . . . 437, 492
Review of Prof. Noel's Article on Histology of Enamel and Dentine. . . . . . . . . . . 95
Review of an Article entitled Dental Hygiene. . . 394
Ross. H. D., D.D.S. . . . . . . . . 34
Rubber Solder. Welch's, . . . . . 336
Rules of Hygiene. . . . . . . . . 188
Saving of Time in Dental Operations. . . . 413
Separating Teeth. . . . . . . . 350
Sesqui Chloride of Chromium in Sensitive Dentine. . 402
Simple Method of Arresting obstinate Epistaxis. . . 520
Simpson. Sir James Y, . . . . . . 141
Skin Grafting Superseded. . . . . . . 524
Somnambulism. . . . . . . . . 188
Southern Dental Association. . . . 46, 109, 477
Southern Dental Association. Second Annual Meeting of, 67
South Carolina State Dental Association. . . 66, 276
Speaking and Singing without a Tongue. . . . 473
Spontaneous Combustion. . . . . . . 332
Spontaneous Ptyalism. . . . . . . 133
Squills as a Poison for Rats. . . . . . . 43
Stomatitis from Fermented Cheese cured by Lemon Juice. 138
Styptic Wool. . . . . . . . . 519
Strychnia as an Antidote to Chloral. . . . 88, 378
Sulphate of Nickel in Neuralgia. . . . . . 141
Sunflower. The Virtue of, . . . . . . 571

Syrup Ether. . . . . . . . 89
Sympathetic Paralysis. . . . . . . 135
Teeth. Irregularities of the, . . . . . 34
Tennessee State Dental Association. . . . . 115
The Oldest Relic of Humanity. . . . . . 141
The Man who may be Expected to Live Long. . . 237
The Career of Dentists. . . . . . . . 524
The Seat of Tumor. . . . . . . . 525
Thackston. W. W. H., D.D.S. . . . . . 240
Therapeutic Power of Oxygen Gas. . . . . 463
Theory of Sleep. . . . . . . . 425
To Detect Manufactured Rum or Brandy. . . . 523
To Reduce Gold and Silver Scraps and Filings to Plate 509
To Remove Foreign Bodies from the Ear. . . . 94
Tobacco and the Parotid Gland. . . . . 383
Transformation of Hydrate of Chloral into Chloroform in the Human Organism. . . . . . . 333
Treatment of Chilblains. . . . . . 423
Treatment of Wounds by Pneumatic Occlusion. . . 521
Tumor Originating in Soft Palate, and Protruding into the Isthmus of the Fauces. . . . . . . 559
Ulceration of Palate in Young Patients. . . . 139
Undeveloped Eye Tooth. . . . . . . 572
Vaccination in the Negro. . . . . . . 423
Valedictory Address. . . . . . . 481
Valuable Combination. . . . . . . 90
Vehicle for Exhibiting Chloral Hydrate. . . . 381
Virginia State Dental Society. . . 275, 354, 383
Vitality and the Vital Forces. . . . . . 337
Vulcanite Combinations. . . . . . . 75
Weight of the Brain. . . . . . . . 185
What Fruit Syrups are Composed of. . . . 332
Words of Cheer. . . . . . . . . 94
Yingling. Geo. S., M.D., D.D.S. . . . . . 402

### TO READERS AND CORRESPONDENTS.

Communications intended for publication, and exchange journals and papers, should be addressed to the EDITOR OF THE AMERICAN JOURNAL OF DENTAL SCIENCE, 259 N. Eutaw St., Baltimore, Md. All letters relating to the business of the journal, or containing cash remittances, to be directed to Messrs. SNOWDEN & COWMAN. Articles for publication should be received by the editor at least three weeks previous to the first day of the month on which the number in which it is designed for them to appear, is issued.

### CONTENTS:

### ORIGINAL COMMUNICATIONS.

ARTICLE I.—The Discovery of Cells with Fibres, Dentine at the Junction of the Enamel and Cementum. By Geo. B. Harriman, D. D. S., ..... 1

ARTICLE II.—Mechanical Dentistry. By J. Z. Hoffer, D.D.S. ..... 5

ARTICLE III.—Recent Views on Digestion. By J. R. Bromwell, D.D.S ..... 14

### SELECTED ARTICLES.

ARTICLE IV.—On the use of Chloroform in Infantile Convulsions. By David Mackintosh, M.D., L.R.C.S ..... 22

ARTICLE V.—Irregularities of the Teeth. By H. D. Ross, D.D.S ..... 34

### MONTHLY SUMMARY.

Hydrate of Chloral, ..... 39

Death from Chloroform. Fatal Tooth Pulling, ..... 39

Almost Complete Ossification of the Human Body, ..... 40

New Operations on the Lower Jaw. Lithotrity, ..... 41

How to Cure a Cold, ..... 41

A Snake in the Stomach, ..... 42

Death Following the Application of Creasote to a Carious Tooth, ..... 43

Squills as a Poison for Rats, ..... 43

Influence of Sewing Machines on Health, ..... 44

Bichloride of Methylene, ..... 44

Doses for Hypodermic Use ..... 45

### EDITORIAL DEPARTMENT.

The Southern Dental Association Meeting at New Orleans, ..... 46

Monument to Dr. Horace Wells, the Discoverer of Aneasthesia, ..... 47

☞ The American Journal of Dental Science will contain fifty pages of reading matter in each number, making a volume of not less than

*Six Hundred* pages;

EXCLUSIVE OF ADVERTISEMENTS.

THE

### AMERICAN JOURNAL OF DENTAL SCIENCE.

F. J. S. GORGAS, M. D., D. D. S., Editor.

The *Journal* is issued on the first of each month and contains not less than *fifty* pages of reading matter in each number, exclusive of advertisements, making a volume of *six hundred* pages per annum.

In each number will be found Original Communications, Correspondence, Selected Articles, Monthly Summary, Bibliographical Notices and Editorial Matter, and every effort will be exerted to render the *Journal* worthy of the patronage of the Dental profession.

A generous co-operation is respectfully solicited from the members of the profession; and as the *Journal* has now a printing office of its own which materially lessens the cost of its publication, it will be issued at the reduced subscription price of

**$2.50 Per Annum in Advance.**

Communications are respectfully solicited on all subjects pertaining to the practice of dentistry, as well as reports of cases occurring in dental practice.

### RATES OF ADVERTISING.

| | 1 MO. | 2 MO. | 3 MO. | 6 MO. | 12 MO. |
|---|---|---|---|---|---|
| 1 PAGE. | $15 00 | $22 50 | $30 00 | $50 00 | $100 00 |
| ½ " | 10 00 | 15 00 | 20 00 | 30 00 | 50 00 |
| ¼ " | 7 00 | 11 00 | 15 00 | 20 00 | 30 00 |
| ⅛ " | 5 00 | 8 00 | 11 00 | 15 00 | 20 00 |

All original communications designed for publication, works for review, new instruments for notice, exchanges, &c., should be directed to the editor, 259 N. Eutaw St., Baltimore, Md.

All advertisements and subscriptions should be addressed to

SNOWDEN & COWMAN,

*Publishers of the Am. Journal of Dental Science.*

86 W. FAYETTE ST. BET. CHARLES & LIBERTY STS.,

BALTIMORE, Md.

